# Criminal Responsibility Scale: Development and Validation of a Psychometric Tool Structured in Clinical Vignettes for Criminal Responsibility Assessments in Brazil

**DOI:** 10.3389/fpsyt.2020.579243

**Published:** 2020-11-27

**Authors:** Leonardo Fernandez Meyer, Cláudia Cristina Studart Leal, Alexandre de Almeida Souza Omena, Katia Mecler, Alexandre Martins Valença

**Affiliations:** Institute of Psychiatry, Federal University of Rio de Janeiro (UFRJ), Rio de Janeiro, Brazil

**Keywords:** criminal responsibility, psychometric tool, factor analysis, forensic psychiatry, assessment, offender, clinical vignettes, insanity defense

## Abstract

Criminal responsibility assessment is undertaken by psychologists or psychiatrists to assess offenders' legal capacities, which vary among countries or regional legislations. There are two psychometric tools (i.e., checklists) validated for criminal responsibility assessment: the Roger Criminal Responsibility Scale, and the rating scale of criminal responsibility for mentally disordered offenders. Despite the existence of psychometric tools structured in clinical vignettes for evaluating legal capacities, none serve the purpose of assessing criminal responsibility. This study aims to validate a novel psychometric tool structured in vignettes for the assessment of criminal responsibility called the “Criminal Responsibility Scale.” We applied the tool to 88 defendants referred for criminal responsibility assessment in a forensic medical institute in the city of Rio de Janeiro, Brazil, from December 2017 to December 2018. The validity of the Criminal Responsibility Scale and subscales were evaluated using confirmatory factor analysis. The two-factor solution proved satisfactory and met the needs for practical application of the tool (Kaiser–Meyer–Oklin = 0.82; *p* < 0.001). Moreover, the inter-rater reliability was evaluated by comparing the tool's final score with that of the expert's conclusion in each case and was found to be satisfactory (*k* = 0.667–1.0), with a resulting cutoff point of 30.50 (±2) and a Youden index of 0.509. Hence, the Criminal Responsibility Scale is an effective psychometric tool for assessments of criminal responsibility that may encourage future research in assessments of legal capacity with clinical vignette-based psychometric instruments.

## Introduction

Criminal responsibility (CR) is the degree of legal liability attributed to a defendant accused of committing an illegal act (1, 2). Ruled out or diminished CR is possible in very specific situations when the defendant is considered not guilty due to an insanity plea (3, 4). In this case, it is claimed that the defendant's mental status at the time of the offense was impaired (1, 2).

CR is a retrospective forensic psychiatric assessment that is based on the individual offender's mental status, interviews with collaterals, analysis of court proceedings, and medical briefs (1, 2). Offenders' CR is determined by their legal capacities (LCs), which correspond to specific psychopathological constructs (1–4). An offender's “legal capacity” refers to his/her ability to act within the framework of the legal system, and this ability is based on whether the offender suffers from psychopathologies as determined by a psychiatrist or psychiatrist (1). The relationship between LCs and clinical symptoms are discussed in the literature, and address the relevant challenges of the forensic technique (4–7).

Article 26 of the Brazilian Penal Code (8) establishes the criteria for determining whether an offender can be considered with ruled out or diminished responsibility, as in the following:

“It is exempt from punishment the agent who, on account of mental illness or incomplete or delayed mental development, was at the time of the action or omission completely incapable of understanding the illicit nature of the fact or of self-determine according to this understanding.

Single paragraph. The sentence may be reduced by one- to two-thirds, if the agent, in virtue of mental disorder or incomplete or delayed mental development, was not completely capable of understanding the illicit nature of the fact or of self-determine according to this understanding.”

Article 26 is based upon the biopsychological criterion, which is applied by the forensic psychiatrist to evaluate the examinee for the presence of psychiatric disorder and clinical impairments at the time of the offense (2). The biopsychological criterion is composed of four components: (i) pathological element (i.e., mental illness or disorder), (ii) causality between the pathological element and the offense, (iii) cognitive and volitional elements, and (iv) the presence of chronological nexus. All four elements must be considered in CR assessments.

The pathological elements have strong compatibility with the nosology for mental disorders present in the International Classification of Mental Disorders (ICD-10) (9). The pathological elements are present in four categories of the ICD-10, namely (a) mental illness (comprising serious psychiatric disorders such as psychotic syndromes, dementia, delirium, and psychosis induced by psychoactive substances that may fully prejudice cognitive or volitional abilities at the time of the offense), (b) mental disorder (comprising psychiatric disorders which partially affect cognitive and volitional elements of imputability, such as personality disorders, paraphilias, mood disorders without psychotic symptoms, and mild intellectual disability), (c) incomplete or delayed mental development (comprising moderate to severe intellectual disability or developmental disorder diagnosis), and (d) addiction (comprising severe substance use disorders) (2).

The causality component of the biopsychological criterion requires a determination of whether the offense is necessarily an expression of a mental disorder (2). The cognitive and volitional elements are referred to as “capacity for understanding” (CU) and “capacity for self-determination” (CD), respectively (2). CU is defined as the capability to fully acknowledge an act's illicit nature, and CD is the aptitude toward self-determination according to this understanding (2).

In Brazil, forensic psychiatrists have three possible conclusions in a defendant's CR assessment (2). The defendant can be considered criminally responsible (i.e., entirely capable of understanding and self-determining according to the act's illicit nature), partially responsible (i.e., partially incapable of understanding and/or self-determining according to the act's illicit nature), and not criminally responsible (i.e., considered entirely incapable of understanding and/or self-determination according to the act's illicit nature) (2). Other countries' legislations also include CU and CD in the assessment of CR, such as the jurisdictions covered by the American Law Institute (ALI) (1, 10). In other jurisdictions, such as those covered by the M'Naghten Rule, only CU is considered accordingly (1, 7).

Notably, the theoretical models defining the psychopathological constructs of CU and CD have been sufficiently developed (1–4, 10–12). The main study on this topic listed 64 elements that describe in detail the psychopathological constructs of CU and CD that are included in article 26 of the Brazilian Penal Code (13). Despite being sufficiently developed, the traditional theoretical models of forensic psychiatry that are applied to CR assessment draw on original legal terminologies that may be outdated. (1, 2, 10, 13). Currently, the pertinence of these theoretical models, in tandem with the need for changes in the wording of legal texts in light of contemporary psychiatric and psychological knowledge, is being deliberated (1, 5, 13, 14). In Canada, the recent discussions to amend the wording of the legislation regarding CR highlight these controversies (15).

Furthermore, theoretical models used in clinical psychiatry have been considered as alternatives to the traditional theoretical models for CR to address biases (6, 7, 10, 16, 17). The low quality of forensic psychiatric reports submitted to the criminal courts and the high rate of inter-examiner disagreement reveal the necessity of improving the CR assessment techniques (18–20). For instance, the writing style, the presence of reasonable forensic arguments underlying the expert's conclusion, the inclusion of a detailed clinical description (i.e., mental status exam), the inclusion of third-party sources of information to the most realistic extent possible (collateral elements) represent quality parameters of the forensic reports (18). The lack of objective parameters for verifying CU and CD are additional issues that need to be considered (19, 20).

The development of specific psychometric tools for examining CR has yielded scientific progress in contemporary forensic psychiatry (21, 22). The Roger Criminal Responsibility Assessment Scale (R-CRAS) and the rating scale of criminal responsibility for mentally disordered offenders (RSCRs) are psychometric tools developed to complement CR assessments (23–25). Both are structured as norm-based inventories with relevant items for CR forensic psychiatric assessment. The R-CRAS consists of 30 items that are typically rated on a six-point scale, and their relevance to psycho-legal issues concerning the insanity plea are represented through 6 cardinal groups: behavioral control, cognitive control, causal nexus, major mental disorder, malingering, and organicity (23, 24). Meanwhile, the RSCRs comprises 18 items regarding criminal motivation: aura before the offense; inducement to the crime; time, place, and object/tool used in the offense; emotion during the offense; shirking responsibility for the offense; concealment of facts during the interview; camouflage; comprehension of the nature of the offense; estimating the consequences; functional impairment; learning or work impairment; impairment of insight; impairment of reality; and impairment of self-control (25).

Psychometric tools developed specifically to assess LCs represent the holistic progression of forensic practice (21, 22, 26–28). Better characterized psychometric tools are structured in vignettes, such as the MacArthur Competence Assessment Tool for Consent to Research (MacCAT-CR), MacArthur Competence Assessment Tool for Treatment (MacCAT-T), MacArthur Criminal Adjudication (MacCAT-CA), and MacArthur Competence Assessment Tool-Fitness to Plead (MacCAT-FP) (26–31). The last tool is commonly used by criminal forensic psychiatrists in Anglo-Saxon countries in support of the experts evaluating a defendant's competency to stand trial (26–30). Clinical vignettes are frequently used in research on the legal theory of competence (26–30) and seem more suitable for LC assessments than psychometric tools that utilize checklists and inventories (i.e., R-CRAS and RSCRs) (23–25).

Clinical vignettes may be frequently used for research on LCs, but their use remains unexplored for CR assessment. There are no psychometric tools to date structured in vignettes to assess CR. Hence, the main objective of this study was to develop and validate a personal interview structured in vignettes to complement the CR assessment, called the “Criminal Responsibility Scale” (CRS).

## Materials and Methods

The CRS is based on the MacArthur-CA clinical vignette structure (26, 28–30) and the main psychopathological theoretical model concerning the assessment of CR in Brazil (13). The main hypothesis tested in our study is that the assessment of the psychopathological constructs of CU and CD can be performed with a structured interview based on clinical vignettes, with questions and answers unrelated to the respective offender or criminal setting. We now describe the elaboration stages of the CRS.

### Theoretical Model for CU and CD

A meticulous review of the forensic psychopathology literature on CU and CD resulted in the identification of 64 related psychopathological constructs (2, 13). According to the literature, LCs can only be assessed through a direct assessment of the examinee (2, 13). Moreover, criminal dynamics, collateral reports, depositions, health treatments, and forensic psychiatric documents provide complementary data for forensic evaluation but they cannot be used to directly assess LCs (2, 13).

CU refers to psychological cognitive elements regarding the perception, grasp, and understanding of reality and natural phenomena (2, 13). In Latin, to understand means to grasp with the mind, to perceive. It also includes a sense of extension, intention, pretension, selection, and a combination of cognitive elements originating from reality (2, 13). Ultimately, to comprehend something means to possess it or grasp it. Classical psychological processes related to comprehending something, such as memory, intelligence, and thinking, are commonly investigated during psychiatric assessments (2, 13).

Conversely, CD refers to self-determination, which entails a sense of delineating, demarcating, regulating, and fixing one's own life. In other words, it involves the notion of a termination, end, or limit to one's involvement in an act (2, 13). Classical psychological processes related to self-determination, such as will, mood, and affect, are commonly investigated during psychiatric assessments. Furthermore, in forensic psychopathology and judicial proceedings, consideration of an individual's actions (or omissions) includes an assessment of the offender's cognitive elements, or CU, followed by an assessment of their self-determination, or CD (2, 13). However, there are legal exceptions to this rule, i.e., factors that attenuate or rule out the illegality of the acts or omissions, such as claims of passion or self-defense (2). Such cases purportedly involve an instinctive, reactive, or impulsive reaction by the offender, regardless of the cognitive elements present before the act or omission (2).

### Structuring of the CRS: Clinical Vignettes

Psychometric tools structured in clinical vignettes are methodologically appropriate for the assessment of LCs (21, 22). The elicitation of histories encompassing themes not correlated to the offender and questions focused on specific psychopathological constructs allow a direct assessment of the examinee's psychopathology (28–31).

Clinical vignettes were prepared for this study that focused on specific psychopathological constructs and the criteria for rating the responses. The MacCAT-CA was used as the main source of ideas for the development of CRS clinical vignettes and the score criteria (26–29). Before using the CRS for the pilot group and final sample, the content for the vignettes was discussed by three forensic psychiatrists who are members of the research team and have considerable experience in CR assessments. The first version of the vignettes included a more detailed scene narrative. During the revision process, we focused on simplifying the text until the description achieved a neutral context, with the ambiance of a common entertainment situation (e.g., a card game). The final clinical vignette portrays the brief history of an offense, as in the following text.

“*I'm going to read a short history for you. Then I'm going to ask your opinion on some points.”*

“*Two people, John and Paul, are playing cards in a room. They are sitting facing each other. Suddenly they begin to argue, and John pushes Paul. Paul falls off the chair, hits his head on the floor, and dies.”*

The psychopathological constructs assessed by the CRS were selected from the previously discussed study on forensic psychopathology in Brazil (13). Among the 64 elements (i.e., psychopathological constructs), a majority were defined as CU and only a few were defined as CD (13), and the distribution of CRS items for the CU and CD subscales were distributed with the same relative proportions.

The CRS included two subscales, titled understanding and self-determination. The purpose of the two subscales was to affirm the statistical validity of the original theoretical model for CU and CD, approved by the Brazilian Penal Code (i.e., biopsychological criteria), and to preserve their original legal and psychopathological meaning in this study (2, 13).

### Composition of the CRS and Scoring Criteria

The psychopathological elements chosen for inclusion in the CRS to represent the CU and CD constructs were debated extensively among the researchers. A few elements were grouped in a single item, given their conceptual similarity. We opted to follow the ordering of the psychopathological elements in the original theoretical model (13).

Based on the vignettes, we drafted at least one question per element. Each question was intended to test a psychopathological construct based on the offender's response, which was recorded verbatim by the examiner and scored based on the scoring criteria referencing specific psychopathological elements (Appendix A).

Additional information or excerpts were added to the vignettes during the interview. The purpose of the excerpts was to modify the individual's initial impression of the vignette by introducing alterations in the psychopathological elements of the respective items. For example, two excerpts were included for item VIII, which consists of three questions. The first two questions (“a” and “b”) pertain to the first excerpt and measure the individual's ability to assimilate or forego values and the coherence of the component elements in the volitional act. The last question (“c”) pertains to the second vignette and assesses the act's legitimacy (Appendix A). A total of four additional excerpts were added to the CRS.

The vignettes' narrative content portrays situations that are easily identified in daily contexts of betting games, disputes, and interpersonal crises, and have a criminal act as the outcome. The researchers discussed various options for the vignettes and ultimately chose to develop more simplified versions representing nominal identification of the active and passive agents of assault to facilitate the examinee's comprehension. Elements of a social, political, regional, religious, economic, or sexual nature were not included in the vignettes or the questions to avoid elements that were not pertinent to the LCs and that could potentially induce assessment bias.

### Final Version of the CRS

The final version of the CRS is summarized in Table 1. Each item explores a specific psychopathological construct, sometimes with more than one question. For didactic reasons, each question should be understood as a subitem of the CRS. The items' order, questions' wording, and subitem scores' criteria were discussed and approved by the research team during the elaboration and testing of the CRS until the tool was finalized.

**Table 1:** Psychopathological constructs of the CRS (items) and the respective number of questions, with division into subscales of understanding and self-determination and Kappa index.

**Psychopathological constructs**	**Number of questions (subitems)**	**Kappa**
Capacity for understand (CU)	Total of 15 questions (subitems)	
(I) The notion of legal good and illegality	2 questions: Items I(A) and I(B)	Item I(A) = 0.680 Item I(B) = 0.926
(II) The notion of duty, legal standard, and criminal definition	1 question: Item II(A)	Item II(A) = 0.970
(III) The notion of potential harm, harmful effect, and overriding criteria	2 questions: Items III(A) and (B)	Item III(A) = 0.740 Item III(B) = 1.000
(IV) The notion of culpability, liability, and responsibility	2 questions: Items IV(A) and (B)	Item IV(A) = 0.827 Item IV(B) = 0.814
(V) Awareness of the act's illegality (prohibitive character) and criminal nature	2 questions: Items V(A) and (B)	Item V(A) = 0.775 Item V(B) = 0.677
(VI) Capacity for value judgment	1 question: Item VI(A)	Item VI(A) = 0.906
(VII) Capacity to weigh alternatives to an act	1 question: Item VII(A)	Item VII(A) = 0.969
(VIII) Capacity to assimilate and forego values. Consistency between the component elements of an act. Awareness of an act's legitimacy	3 questions: Items VIII(A), (B) and (C)	Item VIII(A) = 0.922 Item VIII(B) = 0.954 Item VIII(C)= 0.923
(IX) The notion of an act's harmfulness vs. integrity (censure) and consequences	1 question: Item IX (A)	Item IX(A) = 0.800
Capacity for self-determination (CD)	Total of 4 questions (subitems)	
(X) Perception of social and legal disapproval	2 questions: items X(A) and (B)	Item X(A) = 0.954 Item X(B) = 0.750
(XI) Presence of intent and *animus*	1 question: Item XI(A)	Item XI(A) = 0.717
(XII) Deliberation, decision, and execution	1 question: Item XII(A)	Item XII(A) = 0.922

The final version of the CRS contains 12 items with 19 questions, and the total score varies from 0 to 38. The CU subscale includes 9 items, with a total of 15 questions and a score range of 0 to 30. The CD subscale includes 3 items, with a total of 4 questions (i.e., with a score range of 0–8). The score assigned by the interviewer to each question was “0” (i.e., no acknowledgment of the cited psychopathological elements, or answered with pathological elements), “1” (i.e., partial acknowledgment of at least one of the cited psychopathological elements), or “2” (i.e., assertive acknowledgment of at least one of the cited psychopathological elements).

The final version of the CRS was pilot tested in two different population samples (total *n* = 20). The first group (*n* = 10) consisted of inpatients diagnosed with psychiatric disorders at a clinical psychiatric hospital in Rio de Janeiro city. The second group (*n* = 10) involved controls, i.e., individuals who were not diagnosed with any psychiatric disorder after undergoing a forensic assessment of CR at the same site where this study was being conducted. Only the individuals from the second group (*n* = 10) were included in the final study sample since they met the inclusion criteria.

### Sample

The final sample consisted of 88 participants, who were selected from the individuals referred for a forensic assessment of CR at the Heitor Carrilho Forensic Psychiatry Institute, the only forensic psychiatric institute in the city of Rio de Janeiro. This institution is responsible for all CR exams in Rio de Janeiro State. Participants were selected from among the individuals scheduled for CR assessment from December 2017 to December 2018.

The inclusion criteria were as follows: age 18 years or older, the existence of formal criminal charges brought against the individual by the Public Prosecutor's Office, a court order for forensic assessment of CR, and informed consent for participation. The sample excluded individuals who were unable to communicate verbally, not fluent in Portuguese, represented by insufficient sociodemographic data, or unable to sign informed consent.

### Application of the CRS and Comparison With Forensic Expert's Conclusion

Validation of the CRS included a comparison of its final score with the forensic expert's conclusion, which was considered the gold standard for CR assessment. Our study used three researchers with experience in forensic psychiatric assessment of CR who worked in alternating pairs to select and assess the participants, and oversee the completion of the CRS and sociodemographic questionnaire. First, participants were selected and assessed, and then they underwent the forensic assessment of CR.

The subitem scores of the CRS were rated individually by each pair of researchers. Subitems with divergent scores were discussed by the research pairs on a case-by-case basis to determine the best score in each situation. Subsequently, divergences were examined to verify the agreement between researchers in the application of the CRS.

**Figure 1:**
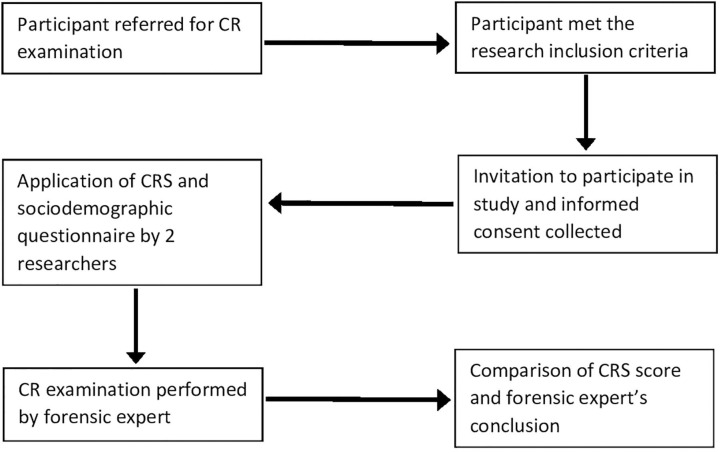
Flowchart for application of the CRS to participants and comparison with forensic expert's conclusion. CR, criminal responsibility; CRS, Criminal responsibility scale.

Finally, the expert's conclusion was compared to the total CRS score (Figure 1). The forensic assessments of CR at Heitor Carrilho Forensic Psychiatry Institute were conducted by a single expert, and there was no communication between the researchers and the expert. Inconclusive cases (*n* = 4) according to the experts were excluded from the final sample, which only included cases accompanied by the conclusion of an assertive forensic expert.

## Results

The participants were defendants, either in custody, or awaiting trial, and were referred for the assessment of CR. A convenience sample was selected from a total of 92 participants, and 4 were excluded due to insufficient data. The final sample consisted of 88 participants, all of whom freely signed the informed consent form to participate in this study.

As shown in Table 2, we analyzed the frequency of psychiatric diagnostic groups (ICD-10), the respective mean values on the CRS, the legal terms applied to participants, and the respective mean values on the CRS. Concerning sociodemographic characteristics, the majority of the sample consisted of males (84.1%), with a mean age of 37.07 years (± 12.65) at the time of the study and a mean age of 34.74 years (± 12.36) at the time of the offense. As for their legal characteristics, 69.8% were awaiting trial and 54.1% had a prior criminal record, with larceny as the most frequent crime. Most of the participants were single (66.3%), lived with their nuclear family and did not have children (53.5%), and were not receiving social security benefits (79.1%). Half (50%) had formal jobs, and the self-reported skin color was black (40.7%), white (34.7%), brown (22%), and yellow (2.3%) according to the Brazilian National Institute of Geography and Statistic (IBGE) classification. Many had not received professional training (68%), and 30.9% had a family income of one monthly minimum wage (~250 USD/month).

**Table 2:** Number and percentage of psychiatric diagnostic groups (ICD-10), legal terminology, and mean CRS scores.

**ICD-10 groups**	***N***	**Percentage (%)**	**Mean CRS**	**Standard deviation**
Intellectual disability	17	19.3	24.29	6.293
Without mental illness	25	28.4	26.76	11.050
Mood/anxiety disorder	3	3.4	29.33	5.508
Personality disorder	5	5.7	32.60	2.702
Psychoactive substance disorder	25	28.4	31.28	3.311
Schizophrenia-like and bipolar mood disorders	13	14.7	24.92	9.412
Total	88	100	27.72	8.128
**Legal terminology**				
Addiction	3	3.4	31.00	0
Delayed mental development	16	18.2	25.44	5.785
Mental illness	18	20.5	24.11	8.436
Mental disorder	5	5.7	32.60	2.702
Without corresponding legal term	46	53.3	29.17	8.744
Total	88	100	27.72	8.128

*ICD 10, international classification of diseases; CRS, Criminal responsibility scale*.

### Completion of the Total CRS

The CRS was completed by recording the participant's responses verbatim, which were always individualized for each subitem, immediately after each question was asked by the interviewer. The interviewer was permitted to repeat the question with an explanation in case the participant has any doubt about the content. The interviewer could use synonyms, but without including personal impressions or inferences when explaining or rewording the question. Each question (subitem) could be repeated up to three consecutive times. Also, the annotated answers were not reworded after being recorded by the interviewer and the score was inspired by the MacCAT-CA (27–30). There are qualitative specificities in the scoring criteria adopted for each subitem (Appendix A).

Similarly, positive reinforcement was given to the participant after answering each item to boost their performance on the CRS. The interviewer could use a bracketed alternative version for each subitem. For example, when reading item I(B) “Do you think John's behavior (the person who pushed Paul) is illegal (or criminal)?” the interviewer can reword the question using the options provided in the brackets. In item I(B), the option is [Do you think what John (the person who pushed Paul) did is illegal (criminal)?]. For item IV(A), “Do you think John is responsible for Paul's death (the pushed person)? Why?” the option is [Do you think John's push caused Paul's death? Why?].

Answers that provided little or no clarity deserve special attention when completing the CRS. Some of these answers represented active symptoms, in which case they received a score of “0.” Symptomatic participants recurrently answered questions with the same phrasal structure and/or content, sometimes with little language variations. These repeated answer patterns were also verified in participants with thinking or intellectual disabilities. However, such situations also occurred when the individual showed little willingness or interest in providing objective responses. In such cases, patterns of responses were obtained that demonstrated the individual's comprehension of the question, despite the lack of objectivity. The speech patterns we identified were: (1) reflexive (or rhetorical), (2) personal, and (3) ideological.

First, the reflexive (or rhetorical) pattern was seen in responses that addressed the questions' contents tangentially but not objectively. In this pattern, the individual intentionally reversed the burden of the response onto the interviewer through the employment of rhetoric. For example, one answer to item IV(A) illustrates this pattern: “But what does being guilty denote in this story? Guilt is relative and subjective. There's something more to explain John's reaction.” The degree of complexity in this response pattern demonstrates satisfactory comprehension of the question's content, does not suggest pathological alterations, and received a score of “2.”

Second, the personal pattern occurred when the individuals used themselves as part of the answer. These cases displayed a victimized attitude, with a predisposition to empathize with or show solidarity toward the clinical vignette's content or characters. For example, one individual responded as follows to item IV(A): “This story sounds like mine. Maybe John is being framed. The story doesn't say whether he was provoked first. It may have been a reaction by John.” Or, “Nobody would push the chair to kill. You'd have to see whether he really intended to kill his friend. It could have been an accident, as in my case.” In such cases, the score assigned to the item was “2,” since it demonstrated the capacity to comprehend the question's content.

Third, the ideological pattern tended to use responses that rival or downplay the content of the vignettes and questions in the CRS. For example, one individual answered I(B) as follows: “It's only illegal in Brazil if you can't afford a lawyer. Jail is only for poor blacks.” Further, in the answer to item IV(A): “The guilty party is always the little guy. We're never going to know what really happened because the story [vignette] is incomplete. Sometimes there are two victims of society.” Thus, the individual tends to use such questions to justify or downplay illegal acts, in the sense of seeking the interviewer's solidarity with the vignette's criminal act, and consequently with their case. In such cases, the score was “2.”

All of these response patterns highlight the complexity of the semantic elements addressed and the ordering of the themes expressed in the responses. Such elements preserve the individual's cognition in deciphering the question's meaning.

### Inter-rater Reliability

When a psychometric test is applied by two or more examiners, it is imperative to verify whether consensus has been achieved with experts' assessments, i.e., whether they interpret and similarly assess the test. One of the most common statistics used in this analysis is Cohen's kappa reliability index. Kappa values from 0.60 to 0.79 reflect moderate levels of agreement, 0.80 to 0.90 indicate a strong level of agreement, and over 0.90 denotes a nearly perfect level agreement. Indices >0.60 are considered satisfactory.

The researchers signified satisfactory inter-rater agreement, with a Cohen's kappa index ranging from 0.677 to 1.0 (Table 1). We used three interviewers who took turns in pairs to complete the CRS and to avoid biases, which was done jointly by both members of the pair.

### Exploratory Factor Analysis

Exploratory factor analysis (EFA) is a practice that summarizes the similarities between variables in a dataset, thereby reducing the dimensions of the data when there are many variables. Some assumptions regarding the dataset need to be stated to use this technique: the relationship between the variables must be linear; the samples must be adequate, that is, with sufficient sample size, a condition verified by the Kaiser–Meyer–Olkin (KMO) test; and the data must be adequate to reduce the dimensions, a condition verified with Barlett's test of sphericity.

The CRS displayed adequate internal consistency, with a KMO value of 0.82, higher than the required minimum of 0.6, proving the sample's adequacy. Barlett's test obtained satisfactory significance (*p* < 0.001), rejecting the null hypothesis that the correlation matrix is an identity matrix meant to utilize the data. Moreover, the KMO and Barlett's test results obtained with the independent analysis of the subitems were identical to those mentioned above.

Similarly, the CRS consists of items and subitems that allow the implementation of two types of EFA. Hence, we first analyzed models that considered the subdivisions (i.e., subitem IA, IB, IIA, etc.) of the data's structure. Then we analyzed models that only considered the entire items (i.e., item I, item II, etc.). The reason for this approach is that selecting entire items ensures greater adequacy in terms of the original theoretical model and between the variables and the factors in the analysis of the results.

The two-factor solution denoted satisfactory values for the adequacy test. The reliability analysis (i.e., Cronbach's alpha) obtained values of 0.72 for factor 1 and 0.72 for factor 2, compared to a required minimum of 0.6. The total variance in the results explained by the model was equal to 36%. The internal reliability values for factors 1 and 2 were acceptable. The CRS showed a Cronbach's alpha of 0.86, and the exclusion of any item from factors 1 and 2 did not improve the performance of this index (Table 3).

**Table 3:** Cronbach's alpha values with the exclusion of items (Factor 1 and Factor 2 data without subdivisions).

	**Mean scale with item excluded**	**Variance of scale with item exclude**	**Total corrected correlation of item**	**Cronbach's alpha with item excluded**
**Factor 1 (CD)**				
Item I	5.87	5.073	0.490	0.686
Item IX	7.36	6.756	0.528	0.672
Item X	6.40	4.221	0.612	0.606
Item XI	7.30	6.805	0.563	0.664
**Factor 2 (CU)**				
Item II	6.20	4.345	0.496	0.702
Item III	5.07	2.359	0.623	0.572
Item IV	4.17	3.687	0.586	0.592

*CD, capacity for self-determination; CU, capacity for understanding*.

**Figure 2:**
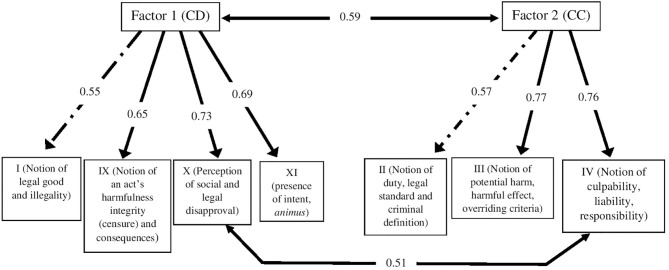
Model for exploratory analysis of the CRS (without subdivision of items). CD, capacity for self-determination; CU, capacity for understanding.

The exploratory factor model, without a subdivision of the items, resulted in a satisfactory fit of the original model (Figure 2). A statistically satisfactory correlation was determined between F1 (CD) and F2 (CU) (*r* = 0.59). Among the three items from the original model for CD, two comprised factor 1, namely items X (i.e., perception of social and legal disapproval) and XI (i.e., intent and *animus*), and both presented significant indices (0.73 and 0.69, respectively). The other components of factor 1, items I (i.e., notion of legal good) and IX (i.e., notion of censure, consequences of the act) were not originally part of the CD, but they also obtained satisfactory indices (0.55 and 0.65, respectively) (Figure 2).

For factor 2 (CU), three items presented satisfactory correlation indices, namely items II (0.57), III (0.77), and IV (0.76). All of these were part of the original model for CU. A correlation was seen between items X (factor 1) and IV (factor 2) (*r* = 0.51). The inclusion of this correlation between items X (factor 1) and IV (factor 2) was essential for developing the model's validation. Despite the weak correlation, the inclusion of this association augmented the final model (Figure 2).

### CRS, Diagnostic Groups, and Legal Terminology

We analyzed correlations between the total CRS and psychiatric diagnostic groups and the legal terminology in the Brazilian Penal Code (Table 4). There were differences in the mean CRS scores between the groups, with the lowest CRS scores reported for the psychotic disorders group (i.e., schizophrenia-like and bipolar mood disorder) and the group with mental illness. The mean score for the group “without mental illness” (26.75 ± 11.050) was lower than for the group with “substance use disorders” (31.28 ± 3.311). This finding may be explained by the presence of conscious simulation (i.e., malingering) in the first group of individuals (Table 4). In the group “without mental disorder related to legal terminology,” the effect of false-negative participants (i.e., individuals with a diagnosis of malingering or conscious simulation) is less evident in the total CRS score (mean 29.17 ± 8.744) (Table 4).

**Table 4:** Mean total CRS score of the sample and disaggregated by ICD-10 groups and legal terminology.

**Diagnostic group (ICD-10)**	**Mean**	***N***	**Standard deviation**
Without mental illness (Z00–04. Z73. Z76.5)	26.76	25	11.050
Intellectual disability (F70–79)	24.29	17	6.293
Mood and anxiety disorders (F32.F33. F41–48)	29.33	3	5.508
Personality disorder (F60–68)	32.60	5	2.702
Substance use disorder (F10–19)	31.28	25	3.311
Schizophrenia-like and bipolar mood disorders (F20–29. F31)	24.92	13	9.412
Total	27.72	88	8.128
**Legal terminology (BPC)**			
Drug addiction	31.00	3	0
Delayed mental development	25.44	16	5.785
Mental illness	24.11	18	8.436
Mental health disturbance	32.60	5	2.702
Without mental disorder related to legal terminology	29.17	46	8.744
Total	27.72	88	8.128

*ICD-10, international classification of diseases; BPC, Brazilian penal code*.

We attempted to test the differences in correlations between the diagnostic groups (ICD-10), legal terminology, and total CRS score. Using the Kruskal–Wallis non-parametric test, a statistically significant difference was found between the groups by psychiatric diagnosis (ICD-10) (*p* = 0.012) and legal terminology (*p* = 0.002).

We found significant differences in the total CRS score among the following psychiatric diagnostic groups: intellectual disability and without mental illness (*p* = 0.039), intellectual disability and personality disorder (*p* = 0.009), intellectual disability and substance use disorder (*p* = 0.001), and substance disorder and schizophrenia-like disorders and a bipolar mood disorder (*p* = 0.032).

We also found the following differences in the total CRS score by legal terminology: delayed mental development and mental health disturbance (*p* = 0.011), delayed mental development and without a corresponding legal term (*p* = 0.003), mental illness and mental health disturbance (*p* = 0.019), and mental illness and without a corresponding legal term (*p* = 0.005).

### ROC Curve and Cutoff Point

ROC curve is used to define a test's cutoff point, such as in a psychometric test. We opted to use the AUC and the statistics for the maximization of sensitivity and specificity (Youden index) to define the ideal cutoff point. Values >0.5 in the Youden index were considered satisfactory for a psychometric test like the CRS.

The total CRS score was compared to the forensic expert's conclusion of criminally responsible, partially responsible, or not criminally responsible. This analysis revealed satisfactory indices for distinguishing between criminally responsible and not responsible individuals, with a cutoff point of 30.50 (±2) (Youden = 0.509). The ROC curve for the other groups (i.e., criminally responsible and partially responsible; not criminally responsible and partially responsible) did not show satisfactory indices.

## Discussion

We describe the statistical validation of the CRS, the first psychometric test structured in vignettes, to complement the assessment of CR. The psychometric properties of the CRS showed satisfactory statistical validity, despite the need for partial adaptation of the original theoretical model. The psychopathological constructs of CU and CD jointly contributed to the original model's validation. A satisfactory correlation between item IV (i.e., the notion of culpability, competence to stand trial, and responsibility) and item X (i.e., perception of legal and social disapproval) reinforces the validity of the alternative hypothesis (Figure 2). This result suggests a correlation between these two psychopathological processes and also how ideological notions of culpability and responsibility (item IV) bridge the recognition of practical notions of legal goods and social benefits (item X). In other words, these two items show, in a direct way, how the interchange of psychopathological performances operate between CU and CD.

The CRS showed satisfactory statistical parameters. The low area under curve (AUC) in our study can be the result of the sample size and does not indicate low quality of the tool. The Cronbach's alpha for the CRS was satisfactory and demonstrated consistency between the items, but still lower than the Cronbach's alpha for the validation of the RSCRs (0.93) and higher than for the R-CRAS (0.63) (24, 25). Differences in the structural design of the psychometric tools and the type of score items adopted explain the difference between the coefficients. The lack of strictly psychopathological variables precluded a detailed comparison of the R-CRAS, RSCRs, and CRS (23–25).

The ROC curve analysis showed satisfactory indices for distinguishing between the criminally responsible vs. not responsible groups. The cutoff may have been influenced by the time between the offense and the CR assessment, exemplified by a mean of 2 years in our sample. This evidence may have modified the psychopathological characteristics of the participants, especially those that presented clinical improvement in the period. A surprising result was the lack of validity of the CRS for the identification of the group with partial responsibility compared to the criminally responsible and not criminally responsible groups. This fact may suggest the technical vulnerability of the partially responsible category in CR assessment, reducing the forensic expert's ability to be assertive about the assessment of CU and CD at the time of the offense (2, 13).

Also, R-CRAS and RSCRs do not directly assess psychopathological constructs, as demanded by law for the assessment of LCs (24, 25). Meanwhile, CRS scrutinizes the inherent psychopathological constructs in CU and CD according to the examinees' performance. Hence, the adoption of objective parameters for scoring answers in CRS enabled the statistical validation of these psychopathological constructs, according to the traditional theoretical model of CR (2, 13). This model presents phenomenological (i.e., subjective) characteristics identical to those adopted by classical psychopathology (13, 32–35).

The inter-examiner agreement of the CRS resulted in a relatively high average value (Table 1). Nevertheless, the characteristics of scientificity inherent in the psychopathological constructs of LCs can be observed in the structure of the CRS and the order of its items and subitems. Moreover, the structure of the clinical vignettes requires rigorous ordering of the psychopathological constructs assessed by the interviewer (21, 22). Other validated psychometric tests in the vignette format for the assessment of LCs reinforce these arguments (27–31).

The nature of the retrospective assessment of CR can introduce differences in the scoring of psychometric tests structured in vignettes, compared to the competence to stand trial via cross-sectional assessment (27–31). This characteristic was seen in the scores obtained with the CRS in each diagnostic group according to the ICD-10 (Table 4). Psychiatric diagnoses with well-defined psychopathological alterations (i.e., schizophrenia, bipolar disorder, and intellectual disability) presented lower CRS scores compared to the other diagnoses (i.e., mood disorder and anxiety disorders related to substance use, personality disorders, and without mental illness) (Table 4). The period between the offense and the forensic assessment (i.e., 2 years on average) may have contributed to this result and the cutoff point obtained in the CRS validation may indicate a potential compromise in the forensic assessment when there are long intervals between the offense and the assessment.

Individuals with a diagnosis of malingering or conscious simulation (Z76.5, by the ICD-10) had the lowest scores on the CRS among the diagnostic groups. This result contributed to the lower score on the CRS in the group without mental illness, compared to the total mean CRS score (Table 1). These individuals (*n* = 8) had the lowest mean CRS scores (i.e., < 10) among all the diagnoses studied. This finding is consistent with the performance of malingerers in clinical and forensic psychometric tests (21, 22).

The comparison of the group with diagnoses according to the ICD-10 and the group with legal terms showed the best performance for the CRS in the latter group. In light of contemporary psychiatry, our study does not support arguments that defend updating psychopathological constructs and current terminologies for diagnostic classification in legal studies (3, 14, 15). The introduction of parameters specifically developed for clinical practice (i.e., the decision-making process) to assess LCs can introduce bias in experts' conclusions, rather than maintaining the technical assumptions of impartiality as preconized (1). Hence, it is crucial to explore concepts of LC in light of their original theoretical models and with psychometric tools specifically developed for this purpose (1, 21, 22). The CRS contributed to the forensic expert's distinction between criminally responsible vs. not responsible individuals despite the time that transpired between the offense and the CR assessment. Special attention should be paid to the group with partial CR in some jurisdictions, as in the Brazilian Penal Code, which did not show statistical reaffirmation in our study (2). Therefore, the forensic expert's conclusion of partial responsibility would appear to be more prone to potential biases when compared to the criminally responsible and not responsible groups. The RSCRs presented satisfactory indices in the identification of criminally responsible, partially criminally responsible, and not criminally responsible individuals, but its structure is not based exclusively on psychopathological elements, as in the forensic assessments of CR (25). Therefore, the identification of partial responsibility in forensic assessments still lacks objective psychopathological criteria.

The legal concepts of CR are defined by specific legislation that vary by country and/or jurisdiction, but there is similarity between them (1). The application of these concepts is strictly related to the legal definitions of LC as defined by specific legislations, the majority of which approve of the CU and CD defendant's CR (1, 2). For instance, as mentioned above, the legal concepts of CR approved by the Brazilian Penal Code are very similar to those approved by the ALI (1, 2, 10). Thus, we argue that the CRS can be applied in countries and jurisdictions that approve the CU and CD for CR assessment.

Our results help answer current questions in forensic psychiatry. The purpose of forensic psychiatry is to support judges and lawyers in developing the necessary legal reasoning for rulings on offenses (1, 2). It is essential to focus on the specificities of concepts used in this area of knowledge, originally from the human sciences, and technically preserve their limits and practical objectives (13, 32–35). To overstep such limits may compromise a judge's performance and the technical quality of forensic evidence used by the parties in the case, and thus irreversibly jeopardize the defendant's trial (1, 2). The use of clinical models not adapted to forensic demands may raise issues that are not pertinent to forensic practice and lead to biases on the part of judges and lawyers (6, 9–11, 13, 15).

These characteristics also show how psychopathology, as a specific area of knowledge, is not limited to psychiatric assessment and is useful in other areas of knowledge, such as law (13, 32–35). Additionally, it is the forensic psychiatrist's responsibility to precisely determine the correspondence between psychopathological elements of law and those applied in psychiatry (13, 32–35).

## Limitations

Our study was based on a sample of defendants in criminal cases (in custody or awaiting trial in liberty) in the state of Rio de Janeiro, Brazil. Similar studies in other states of Brazil are needed for this purpose. Furthermore, regional and cultural factors can affect the validity of the CRS. In this study, the CRS was applied by two researchers taking turns in a three-person team. The results were compared to the reports by two official forensic experts at the Heitor Carrilho Forensic Psychiatry Institute. The application of the CRS in other forensic institutes, both in Brazil and in other countries, is essential for improving CRS applicability and the research in this field.

The research on the CRS by other forensic institutes in Brazil and other jurisdictions is crucial for its refinement. The translation and validation of the CRS in other languages for application in other jurisdictions will also be important for continuing the research in this field.

## Conclusion

The CRS revealed objective criteria for determining CU and CD in the assessment of CR. The phenomenological nature of these LCs precludes an immediately objective determination and requires elements from psychopathology for its objective determination (2, 13, 32–35). Our study helps answer the following key question: What are the appropriate criteria of scientificity for subjective psychopathological constructs? Our results point to the analysis of semantic elements that proved to be safe parameters with required standards of scientificity for subjective psychopathological constructs. The analysis of semantic elements may be expanded by future research in this area. Additionally, the CRS proved to be a useful complementary method in forensic assessments of CR, without ruling out an analysis by a forensic expert.

## Data Availability Statement

The original contributions presented in the study are included in the article/Supplementary Materials, further inquiries can be directed to the corresponding author/s.

## Ethics Statement

The studies involving human participants were reviewed and approved by Committee of Ethical in Research of Federal University of Rio de Janeiro. The patients/participants provided their written informed consent to participate in this study.

## Author Contributions

LM was responsible for project conceptualization, data collection, analysis, literature research, English translation, methodology, organization, statistical analysis, article writing, and review. CL was responsible for data collection, project administration, and article revision. AO was responsible for supporting the data collection and project administration procedures. KM was responsible for project administration, data analysis, translation, and review. AV was responsible for supporting the project conceptualization, article writing and review, and project supervision. All authors contributed to the article and approved the submitted version.

## Conflict of Interest

The authors declare that the research was conducted in the absence of any commercial or financial relationships that could be construed as a potential conflict of interest.

## References

[1] Rosner R Scott C Principles and Practice of Forensic Psychiatry. 3rd editors. Boca Raton, FL: CRC Press (2017).

[2] Taborda JG. Criminal justice system in Brazil: functions of a forensic psychiatrist. Int J Law Psychiatry. (2001) 24:371–86. 10.1016/S0160-2527(01)00073-5 11521415

[3] Meynen G. A neurolaw perspective on psychiatric assessments of criminal responsibility: decision-making, mental disorder, and the brain. Int J Law Psychiatry. (2013) 36:93–9. 10.1016/j.ijlp.2013.01.001 23433730

[4] Slovenko R. The mental disability requirement in the insanity defense. Behav Sci Law. (1999) 17:165–80. 10.1002/(SICI)1099-0798(199904/06)17:2<165::AID-BSL337>3.0.CO;2-Y 10398328

[5] Buchanan A. Who needs capacity? Int J Law Psychiatry. (2015) 40:1–5. 10.1016/j.ijlp.2015.04.001 25939285

[6] Niveau G Sozonets E. Criminal responsibility assessment in Switzerland: changes and continuity. Eur Psychiatry. (2001) 16:483–90. 10.1016/S0924-9338(01)00610-1 11777739

[7] Miller L Psychological evaluations in the criminal justice system: basic principles and best practices. Aggress Violent Behav. (2013) 18:83–91. 10.1016/j.avb.2012.10.005

[8] Brasil Decreto-lei No. 2.484/40 (Código Penal). (1940). Available online at: http://www.planalto.gov.br/ccivil_03/decreto-lei/del2848compilado.htm (accessed November 03, 2020).

[9] Bramer GR International statistical classification of diseases and related health problems - tenth revision. World Heal Stat Q. (1988) 41:32–6. Available online at: https://apps.who.int/iris/handle/10665/42980 3376487

[10] American Academy of Psychiatry and the Law (AAPL) Janofsky JS Hanson A Candilis PJ Myers WC Zonana H AAPL practice guideline for forensic psychiatric evaluation of defendants raising the insanity defense. J Am Acad Psychiatry Law. (2014) 42:S3–76. Available online at: http://www.jaapl.org/content/42/4_Supplement/S3.abstract 25492121

[11] Bloechl AL Vitacco MJ Neumann CS Erickson SE. An empirical investigation of insanity defense attitudes: exploring factors related to bias. Int J Law Psychiatry. (2007) 30:153–61. 10.1016/j.ijlp.2006.03.007 17166589

[12] Meynen G. An ethical framework for assessments of criminal responsibility: applying Susan Wolf's account of sanity to forensic psychiatry. Int J Law Psychiatry. (2012) 35:298–304. 10.1016/j.ijlp.2012.04.011 22627087

[13] Chalub M Introdução à Psicopatologia Forense: Entendimento e Determinação (Translation: Introduction to Forensic Psychopathology: Understanding and Determination). 1st Edition. Rio de Janeiro: Companhia Editora Forense (1981).

[14] Burrows M Reid WH. Law and psychiatry psychiatric aspects of criminal responsibility: Insanity and mitigation. J Psychiatr Pract. (2011) 17:429–31. 10.1097/01.pra.0000407967.80345.ac 22108401

[15] Lacroix R O'Shaughnessy R McNiel DE Binder RL. Controversies concerning the Canadian not criminally responsible reform act. J Am Acad Psychiatry Law. (2017) 45:44–51. Available online at: https://www.scopus.com/inward/record.uri?eid=2-s2.0-85014765655&partnerID=40&md5=cbaeaf4c650c9d5a5fdbdeeb0d0db8f8 28270462

[16] Kalis A Meynen G. Mental disorder and legal responsibility: the relevance of stages of decision making. Int J Law Psychiatry. (2014) 37:601–8. 10.1016/j.ijlp.2014.02.034 24694295

[17] Parmigiani G Mandarelli G Meynen G Tarsitani L Biondi M Ferracuti S. Free will, neuroscience, and choice: towards a decisional capacity model for insanity defense evaluations. Riv Psichiatr. (2017) 52:9–15. 10.1708/2631.27049 28287192

[18] Robinson R Acklin MW. Fitness in paradise: quality of forensic reports submitted to the Hawaii judiciary. Int J Law Psychiatry. (2010) 33:131–7. 10.1016/j.ijlp.2010.03.001 20483159

[19] Fuger KD Acklin MW Nguyen AH Ignacio LA Gowensmith WN. Quality of criminal responsibility reports submitted to the Hawaii judiciary. Int J Law Psychiatry. (2014) 37:272–80. 10.1016/j.ijlp.2013.11.020 24326082

[20] Kacperska I Heitzman J Bak T Leśko AW Opio M. Reliability of repeated forensic evaluations of legal sanity. Int J Law Psychiatry. (2016) 44:24–9. 10.1016/j.ijlp.2015.08.028 26346685

[21] El-Shenawy OE Traditional psychological tests usage in forensic assessment. Forensic Leg Investig Sci. (2019) 3:1–5. 10.24966/FLIS-733X/100020

[22] Simms LJ Classical and modern methods of psychological scale construction. Soc Personal Psychol Compass. (2008) 2:414–33. 10.1111/j.1751-9004.2007.00044.x

[23] Rogers R Seman W Clark CR. Assessment of criminal responsibility: Initial validation of the R-CRAS with the M'Naghten and GBMI standards. Int J Law Psychiatry. (1986) 9:67–75. 10.1016/0160-2527(86)90017-8 3793347

[24] Rogers R Sewell KW. The R-CRAS and insanity evaluations: A re-examination of construct validity. Behav Sci Law. (1999) 17:181–94. 10.1002/(SICI)1099-0798(199904/06)17:2<181::AID-BSL338>3.0.CO;2-4 10398329

[25] Cai W Zhang Q Huang F Guan W Tang T Liu C. The reliability and validity of the rating scale of criminal responsibility for mentally disordered offenders. Forensic Sci Int. (2014) 236:146–50. 10.1016/j.forsciint.2013.12.018 24529786

[26] Hoge SK Bonnie RJ Poythress N Monahan J Eisenberg M Feucht-Haviar T. The MacArthur adjudicative competence study: development and validation of a research instrument. Law Hum Behav. (1997) 21:141–79. 10.1023/A:1024826312495 9146101

[27] Appelbaum PS. Assessment of patients' competence to consent to treatment. N Engl J Med. (2007) 357:1834–40. 10.1056/NEJMcp074045 17978292

[28] Hoge SK Poythress N Bonnie R Eisenberg M Monahan J Feucht-Haviar T. Mentally III and non-mentally III defendants' abilities to understand information relevant to adjudication: a preliminary study. Bull Am Acad Psychiatry Law. (1996) 24:187–97. 8807159

[29] Otto RK Poythress NG Edens JF Nicholson RA Monahan J Bonnie RJ Psychometric properties of the MacArthur competence assessment tool- criminal adjudication. Psychol Assess. (1998) 10:435–43. 10.1037/1040-3590.10.4.435

[30] Akinkunmi AA. The MacArthur competence assessment tool - fitness to plead: a preliminary evaluation of a research instrument for assessing fitness to plead in England and Wales. J Am Acad Psychiatry Law. (2002) 30:476–482. 12539898

[31] Zapf PA Roesch R. An investigation of the construct of competence: a comparison of the FIT, the MacCAT-CA, and the MacCAT-T. Law Hum Behav. (2005) 29:229–52. 10.1007/s10979-005-2194-4 15912726

[32] Parnas J Sass LA Zahavi D. Rediscovering psychopathology: the epistemology and phenomenology of the psychiatric object. Schizophr Bull. (2013) 39:270–7. 10.1093/schbul/sbs153 23267191 PMC3576163

[33] Jaspers K. The phenomenological approach in psychopathology. Br J Psychiatry. (1968) 114:1313–23. 10.1192/bjp.114.516.1313 5702272

[34] Fuchs T The phenomenology and development of social perspectives. Phenomenol Cogn Sci. (2013) 12:655–83. 10.1007/s11097-012-9267-x

[35] Meyer LF Taborda JG da Costa FA Soares AL Mecler K Valença AM. Phenomenological aspects of the cognitive rumination construct. Trends Psychiatry Psychother. (2015) 37:20–6. 10.1590/2237-6089-2014-0025 25860563

