# Supplementary material for: Criminal Responsibility Scale: Development and Validation of a Psychometric Tool Structured in Clinical Vignettes for Criminal Responsibility Assessments in Brazil
Source: Front Psychiatry. 2020 Nov 27;11:579243. doi: 10.3389/fpsyt.2020.579243 (PMC7728607; doi:10.3389/fpsyt.2020.579243)
Supplement: Supplementary file 1 [file Data_Sheet_1.PDF]

Appendix A

CRIMINAL RESPONSIBILITY SCALE

"I'm going to read a short history for you. Then I'm going to ask your opinion on some points."

Vignette 1

"Two people, John and Paul, are playing cards in a room. They are sitting facing each other. Suddenly they begin to argue, and John pushes Paul. Paul falls off the chair, hits his head on the floor, and dies."

CAPACITY FOR UNDERSTANDING

I. The notion of Legal goods and illegality

(A) "*Do you think that there is something strange in the story I told you? (after the first answer) Why?" Or ["Do you think there's something unusual about the story that I told you?"]*

Answer:

Score: (0) (1) (2)

CRITERION I (A)

(2) Acknowledges assertively at least 1 of the following elements: occurrence of a crime (death of an individual or assault on physical integrity); violation of the social order and rules of proper social and community life.

(1) Partially acknowledges at least 1 of the following elements: occurrence of a crime (death of an individual or assault on physical integrity); violation of the social order and rules of proper social and community life.

(0) Fails to acknowledge the above elements. Responds with symptoms of active psychiatric disorder, without answering satisfactorily.

(B) *"Do you think John's behavior (the person who pushed Paul) is illegal (or criminal)? (after the first answer) why? Or ["Do you think what John (the person who pushed Paul) did is illegal (criminal)?"]*

Answer:

Score: (0) (1) (2)

CRITERION I (B)

(2) Acknowledges assertively **at least 1** of the following elements: damage to victim's legally protected property: death, aggression and/or damage to another, withdrawal of another fundamental right (life, physical and psychological well-being, material property), without chance of defense.

(1) Partially acknowledges **at least 1** of the following elements: damage to victim's legally protected property: death, aggression and/or damage to another, withdrawal of another fundamental right (life, physical and psychological well-being, material property), without chance of defense.

(0) Fails to acknowledge the above elements. Responds with symptoms of active psychiatric disorder, without answering satisfactorily.

Item I total score:

II. The notion of duty, legal standard, and criminal definition

(A) *"In your opinion, what should John do after he pushed Paul and realized the accident?" Or ["What did John have to do after he pushed Paul and saw him faint (disagreed)?"]*

Answer:

Score: (0) (1) (2)

CRITERION II (A)

(2) Acknowledges assertively **at least 1** of the following elements: need to provide or seek assistance for the victim; call the police or authority; seek help from others.
(1) Partially acknowledges **at least 1** of the following elements: need to provide or seek assistance for the victim; call the police or authority; seek help from others.
(0) Fails to acknowledge the above elements. Responds with symptoms of active psychiatric disorder, without answering satisfactorily.

Item II total score:

III. The notion of potential harm, harmful effect, and overriding criteria

(A) *"In your opinion, was John able to predict (or imagine) that he could hurt Paul with a push? (after the first answer) Why?" Or ["Do you think John could have guessed (or had an idea) that Paul would get hurt with the push?"]*
Answer:

Score: (0) (1) (2)

CRITERION III (A)
(2) Acknowledges assertively that the push could hurt the victim.
(1) Partially acknowledges that the push could hurt the victim.
(0) Fails to acknowledge the above elements. Responds with symptoms of active psychiatric disorder, without answering satisfactorily.

(B) *"Can you give me an example of another negative consequence (bad consequence) that could have happened to Paul (the pushed person), different from death?" Or ["Can you give me an example of another problem that Paul (the pushed person) could have suffered from the push?"]*
Answer:

Score: (0) (1) (2)

CRITERION III (B)

(2) Acknowledges assertively **at least 1** possible negative consequence to the victim (harmful consequence, in addition to death) resulting from the agent's action (e.g., causing trauma, injury, bleeding, fractures).

(1) Partially acknowledges **at least 1** possible negative consequence to the victim (harmful consequence, in addition to death) resulting from the agent's action, (e.g., causing trauma, injury, bleeding, fractures).

(0) Fails to acknowledge the above elements. Responds with symptoms of active psychiatric disorder, without answering satisfactorily.

Item III total score:

IV. The notion of culpability, liability, and responsibility

(A) "*Do you think John is responsible for Paul's death (the pushed person)? (after the first answer) Why?" Or ["Do you think John's push caused Paul's death?"]*

Answer:

Score: (0) (1) (2)

CRITERION IV (A)

(2) Acknowledges assertively that John is the agent responsible for Paul's fall and/or death.

(1) Acknowledges partially that John is the agent responsible for Paul's fall and/or death.

(0) Fails to acknowledge the above elements. Responds with symptoms of active psychiatric disorder, without answering satisfactorily.

(B) *"Do you think John should be brought to trial? (after the first answer) Should he be found guilty or not guilty? Why should he be found guilty?" Or ["Do you think John should be brought to justice for pushing Paul? Why?"]*

Answer:

Score: (0) (1) (2)

**CRITERION IV (B)**
(2) Acknowledges assertively that John is responsible for his action and/or should be brought to trial.
(1) Acknowledges partially that John is responsible for his action and/or should be brought to trial.
(0) Fails to acknowledge the above elements. Responds with symptoms of active psychiatric disorder, without answering satisfactorily.

Item IV total score:

V. Awareness of the act's illegality (prohibitive character) and criminal nature

(A) "*Do you think pushing a person is a crime, even if nobody dies or gets hurt?" Or ["Do you think pushing a person can be considered a crime?"]*

Answer:

Score: (0) (1) (2)

**CRITERION V (A)**
(2) Acknowledges assertively that pushing can be considered a crime when: there is damage, injury, or harm to the victim, promotion of social disorder, violation of social norms, confrontation of legal determination, and it does not occur by self-defense. Or, the push can NOT be considered a crime in specific contexts (e.g., football games).
(1) Acknowledges partially that pushing can be considered a crime when: there is damage, injury or harm to the victim, promotion of social disorder, violation of social norms, confrontation of legal determination, and it does not occur by self-defense. Or, the push can NOT be considered a crime in specific contexts (e.g., football games).

(0) Fails to acknowledge the above elements. Responds with symptoms of active psychiatric disorder, without answering satisfactorily.

(B) *"Can you give me a real-life example to explain your previous answer (different from what I told you)?"Or ["Do you think a push can be considered a crime?"]*
Answer:

Score: (0) (1) (2)

CRITERION V (B)
(2) Is able to formulate assertively an example that illustrates the answer previously given (no value judgment or ethical-moral).
(1) Is partially able to formulate an example that illustrates the answer previously given (no value judgment or ethical-moral).
(0) Fails to acknowledge the above elements. Responds with symptoms of active psychiatric disorder, without answering satisfactorily.

Item V total score:

VI. Capacity for value judgment

(A) *"What do you think about John pushing Paul out of the chair as in the narrated situation? (After the first answer) Why?"*
Answer:

Score: (0) (1) (2)

CRITERION VI (A)
(2) Acknowledges assertively **at least 1** element appropriate to the situation: solidarity, compassion, ethical-moral values, social and criminal reproach, and impossibility of reparation.
(1) Acknowledges partially **at least 1** element appropriate to the situation: solidarity, compassion, ethical-moral values, social and criminal reproach, and impossibility of reparation.
(0) Fails to acknowledge the above elements. Responds with symptoms of active psychiatric disorder, without answering satisfactorily.

Item VI total score:

VII. Capacity to weigh alternatives to an act

(A) *"Can you tell me an example of how to resolve this argument, without John pushing Paul? (After the first answer) Why did you choose this example?" Or [“Can you tell how John and Paul could resolve their argument without the push? Why did you choose this example?”]*
Answer:

Score: (0) (1) (2)

CRITERION VII (A)

(2) Acknowledges assertively **at least 1** viable solution to the proposed situation that does not involve hostility, violence, or delinquency.

(1) Acknowledges partially **at least 1** viable solution to the proposed situation that does not involve hostility, violence, or delinquency.

(0) Fails to acknowledge the above elements. Responds with symptoms of active psychiatric disorder, without answering satisfactorily.

Item VII total score:

Vignette 2

“*Let’s suppose that the argument between John and Paul had started because John saw a card under Paul’s foot (the person who was pushed). So, John thought Paul had cheated, and he had lost most rounds in the card game as a result.*”

VIII. Capacity to assimilate and forego values. Consistency between the component elements of an act. Awareness of an act’s legitimacy.

(A) “*What do you think of this new situation in the story?” Or [“What is your opinion about this new story?”]*
Answer:

Score: (0) (1) (2)

CRITERION VIII (A)

(2) Capable of assertively including **at least 1**: element/value (rational or affective) not used previously and linked to the proposed new situation OR forgoing previously included values.

(1) Capable of partially including **at least 1**: element/value (rational or affective) not used previously and linked to the proposed new situation OR forgoing previously included values.

(0) Incapable of acknowledging any of the above elements. Offers alternatives with symptoms of active psychiatric disorder, without providing satisfactory answers.

(B) *Do you think there is another possibility that explains the playing card under Paul's foot, other than that he was cheating? Or ["How do you think the playing card ended up under Paul's foot (the pushed person)?"]*

Answer:

Score: (0) (1) (2)

CRITERION VIII (B)

(2) Capable of assertively stating **at least 1** plausible alternative to the interpretation suggested in the vignette (cheating).

(1) Capable of partially stating **at least 1** plausible alternative to the interpretation suggested in the vignette (cheating).

(0) Incapable of acknowledging any of the above elements. Offers alternatives with symptoms of active psychiatric disorder, without providing satisfactory answers.

Vignette 3

"Let's suppose that Paul (the person who was pushed) had threatened to attack John with a knife during the most heated part of the card game, just before he was pushed."

(C) "*What do you think of this new situation in the story? (after the first answer) Why?"*

Answer:

Score: (0) (1) (2)

CRITERION VIII (C)

(2) Capable of assertively pointing to **at least 1** element pertaining to: the act's legitimacy (self-defense); the existence of risk (imminent threat) to life, with concordance between the act (pushing) and cause (threat of death).

(1) Capable of partially pointing to **at least 1** element pertaining to: the act's legitimacy (self-defense); the existence of risk (imminent threat) to life, with concordance between the act (pushing) and cause (threat of death).

(0) Incapable of acknowledging any of the above elements. Offers alternatives with symptoms of active psychiatric disorder, without providing satisfactory answers.

Item VIII total score:

IX. The notion of an act's harmfulness versus integrity (censure) and consequences

(A) *"For you, what could make John forego pushing Paul out of the chair? (after the first answer) Why?" Or ["What do you think could change John's decision to push Paul?"]*
Answer:

Score: (0) (1) (2)

CRITERION IX (A)

(2) Capable of assertively pointing to **at least 1** of the following elements: notion of harmfulness and potentially harmful action, possibility of avoiding negative outcomes, feelings of coercion, internal correction, and rectitude.

(1) Capable of partially pointing to **at least 1** of the following elements: notion of harmfulness and potentially harmful action, possibility of avoiding negative outcomes, feelings of coercion, internal correction, and rectitude.

(0) Incapable of acknowledging any of the above elements. Offers alternatives symptoms of active psychiatric disorder, without providing satisfactory answers.

Item IX total score:

THE UNDERSTANDING SUBSCALE TOTAL SCORE:

CAPACITY FOR SELF-DETERMINATION

X. Perception of social and legal disapproval

(A) *“What do you think John felt after he pushed Paul and realized the consequence of the accident? (after the first answer) Why?”” Or [“In your opinion, what was John's feeling after pushing Paul and realizing he died?]*
Answer:

Score: (0) (1) (2)

CRITERION X (A)
(2) Capable of assertively pointing to **at least 1** of the following elements: moral disapproval (modesty, indifference, rectitude) and social disapproval (risk to the social order); feeling of equality, altruism, and solidarity; breaking of social discipline.
(1) Capable of partially pointing to **at least 1** of the following elements: moral disapproval (modesty, indifference, rectitude) and social disapproval (risk to the social order); feeling of equality, altruism, and solidarity; breaking of social discipline.
(0) Incapable of acknowledging any of the above elements. Offers alternatives with symptoms of active psychiatric disorder, without providing satisfactory answers.

Vignette 4
"Let's imagine that John and Paul were playing cards alone. So nobody would know about the push (i.e., be a witness) and John could say that Paul fell to the ground alone if they accused him of killing the victim. ”

(B) “*Do you think that being in an environment without other people might have influenced John to push Paul from the chair?” Or [If there were more people present, do you think John would have pushed Paul?]*
Answer:

Score: (0) (1) (2)

**CRITERION X (B)**
(2) Acknowledges assertively that being in an environment without other people makes it difficult for individuals to incriminate themselves (absence of witnesses).<br>(1) Partially acknowledges that being in an environment without other people makes it difficult for individuals to incriminate themselves (absence of witnesses)<br>(0) Fails to acknowledge the above elements. Responds with psychiatric disturbances, without answering satisfactorily.

Item X total score:

XI. Presence of intent and animus

(A) *“In your opinion, what were John's intentions in pushing Paul? (after the first answer) Why?" Or [“What were John's goals in pushing Paul?”]*
Answer:

Score: (0) (1) (2)

CRITERION XI (A)
(2) Capable of assertively pointing to **at least 1** of the following elements: instinctive motivations (dispositions and tendencies), state of animus, proportionality of the action practiced with the intended purpose.<br>(1) Capable of partially pointing to **at least 1** of the following elements: instinctive motivations (dispositions and tendencies), state of animus, proportionality of the action practiced with the intended purpose.<br>(0) Incapable of acknowledging any of the above elements. Offers alternatives with symptoms of active psychiatric disorder, without providing satisfactory answers.

Item XI total score:

XII. Deliberation, decision, and execution

(A) *“In your opinion, was John's best possible choice to push Paul? “Or [Could John have acted differently in this situation to achieve his goal?”]*
Answer:

Score: (0) (1) (2)

CRITERION XII (A)

(2) Capable of assertively pointing to **at least 1** of the following elements: possibility of avoiding pain; feeling of disgust or regret; ability to bear the consequences of choice; notion of effectiveness/efficiency of action.

(1) Capable of partially pointing to **at least 1** of the following elements: possibility of avoiding pain; feeling of disgust or regret; ability to bear the consequences of choice; notion of effectiveness/efficiency of action.

(0) Incapable of acknowledging any of the above elements. Offers alternatives with symptoms of active psychiatric disorder, without providing satisfactory answers.

Item XII total score:

THE SELF-DETERMINATION SUBSCALE TOTAL SCORE

CRIMINAL RESPONSIBILITY SCALE TOTAL SCORE
